# *Luffy* gen. nov., a new genus of Staphylinina (Coleoptera, Staphylinidae, Staphylininae), remarkable for understanding the *Eucibdelus* lineage

**DOI:** 10.3897/zookeys.1281.198593

**Published:** 2026-06-05

**Authors:** Fang-Shuo Hu, Alexey Solodovnikov

**Affiliations:** 1 Natural History Museum of Denmark, University of Copenhagen, Universitetsparken 15, Copenhagen 2100, Denmark Natural History Museum of Denmark, University of Copenhagen Copenhagen Denmark

**Keywords:** New species, *Ocypus*-group, Oriental Region, rove beetles, Staphylinini, Staphylinina, taxonomy

## Abstract

We describe and illustrate a new genus of Staphylinina, *Luffy***gen. nov**., with two new species: *L. schillhammeri***sp. nov**. from Yunnan, China, and *L. nika***sp. nov**. from northern Laos. The new genus is placed within the *Ocypus*-group and is hypothesized to be sister to the *Eucibdelus* lineage. The synapomorphic characters of the *Eucibdelus* lineage are discussed, and its diagnosis is updated.

## Introduction

The subtribe Staphylinina is a diverse group of large rove beetles of generalist and specialist predators present in various terrestrial biomes (Hu et al. 2025a). While the subtribe currently comprises 47 genera and over 900 species globally (Zhao et al. 2025), its actual species richness and the monophyly-based lineage diversity at the generic level remain insufficiently explored, leaving the internal classification of the group in a state of flux.

Within Staphylinina, the *Eucibdelus* lineage represents a primarily Oriental group, with only a few species extending into the eastern Palaearctic region. Most members exhibit specialized arboreal or floricolous habits (Schillhammer 2012) and ambush hunting behaviour (Hu et al. 2025a). The systematic status of this lineage has long remained unstable. Sharp (1889) first proposed Eucibdelini in connection with his description of the genus *Miobdelus* Sharp. Although he did not provide a list of included genera, he compared *Miobdelus* with *Eucibdelus* Kraatz, *Philetaerius* Sharp, *Phytolinus* Sharp, *Rhynchocheilus* Sharp, and *Trichocosmetes* Kraatz, probably implying that all of them formed Eucibdelini. He further suggested that *Philetaerius* represented a transitional form between the “*Philonthus* group of genera” and Eucibdelini, whereas *Miobdelus* represented a transition between Eucibdelini and *Ocypus* Leach. This suprageneric taxon was largely ignored in subsequent major works. For example, although Cameron (1932) and Scheerpeltz (1940) redescribed a number of related genera, neither author adopted nor discussed Eucibdelini.

Only after a century, Hayashi (1997, 1999a) made further attempts to define the *Eucibdelus* lineage (“*Eucibdelus* group” as he called it in his description of the monotypic genus *Paraphytolinus* Hayashi). However, his definitions of Eucibdelini were later criticized by Schillhammer (2001), who argued that they were based on limited material and highly variable characters. In a subsequent study, Hayashi (2005) downgraded Eucibdelini to subtribal level (Eucibdelina), suggested the inclusion of *Philetaerius* and *Leistotrophus* Perty (misspelled as *Leistrophus*), and proposed a close relationship of Eucibdelina with Anisolinina. Later, Schillhammer (2001) did not include *Miobdelus* in the list of genera of the *Eucibdelus* group but without further explanation on this genus.

Smetana and Davies (2000) and Smetana (2003) omitted the *Eucibdelus* lineage from their systematic revisions of the northern temperate and especially Chinese Staphylinina, respectively, which they called “*Staphylinus*-complex” because they argued that members of the *Eucibdelus* lineage were easily distinguishable from the rest of the complex by their dilated front tibiae and specialized arboricolous-floricolous habits. However, subsequent observations have revealed that these character states are not universal across all species or even all genera within the lineage (Schillhammer 2023).

Recent molecular and morphological phylogenetic studies of the tribe Staphylinini have substantially reshaped our understanding of relationships within Staphylinina. The former subtribe Eucibdelina was synonymized with Staphylinina based on molecular evidence (Chani-Posse et al. 2018), a conclusion further supported by larval morphology (Li and Tang 2024; Hu et al. 2025b) and total evidence phylogenetic analyses (Brunke and Smetana 2019). Brunke and Smetana (2019) recognized three informal groups within the subtribe: the *Creophilus*-group, the *Platydracus*-group, and the *Ocypus*-group, the latter including the *Eucibdelus* lineage. Furthermore, Brunke and Smetana (2020) provided a summary of the genera included in the *Ocypus*-group, with all genera associated with the *Eucibdelus* lineage placed there. The most recent phylogenomic study by Zhao et al. (2025) corroborated the monophyly of these three groups and in particular demonstrated that both *Philetaerius* and *Leistotrophus* belong to the *Platydracus*-group (Zhao et al. 2025). However, even that study left the placement of several genera (e.g. *Acupronotes* Brunke & Smetana, *Apostenolinus* Bernhauer, and *Staphylinus* Linnaeus) in either *Ocypus*- or *Platydracus*-groups uncertain.

Despite these advances, the composition, monophyly and sister group relationships of the *Eucibdelus* lineage has never been rigorously assessed due to limited taxon sampling of this lineage in formal phylogenetic analyses or character discussions. To date, only a single species of *Eucibdelus* has been included in the morphological phylogenetic datasets (Li and Tang 2024; Hu et al. 2025b), and only one species of *Rhynchocheilus* has been sequenced for a molecular phylogenetic analysis (Brunke and Smetana 2019). Most recently, in the revision of the genus *Eucibdelus*, Schillhammer (2023) suggested that the *Eucibdelus* lineage can be characterized by the absence of a semimembranous extension of the labrum and the frequent presence of dilated protibiae and patellate protarsi.

The genus- and species-level taxonomic knowledge of the lineage has gradually expanded through revisions or standalone species descriptions. In a brief overview of the lineage, Schillhammer (2001) described several new genera and species. Other studies have contributed additional species descriptions and taxonomic notes across multiple genera (e.g. Hayashi 1998a, 1998b, 1998c, 1999b; Schillhammer 2004; Tang and Cheng 2020; Hayashi 2021; He et al. 2021; Tang et al. 2021; Schillhammer 2023, 2025; Huang et al. 2025).

Lack of focused phylogenetic work for the *Eucibdelus* lineage, continuing high rate of taxonomic discovery and our observations contradicting in some details the latest morphological diagnosis of this group proposed by Schillhammer (2023), suggest that our understanding of this lineage is far from being settled. A remarkable new genus of Staphylinina from the Oriental region described in this paper clearly proves this. This genus exhibits a unique combination of characters intermediate between the *Eucibdelus* lineage and other members of the *Ocypus*-group. Based on this discovery, we reassess the putative synapomorphies of the *Eucibdelus* lineage and discuss the systematic position of this lineage and the new genus within Staphylinina.

## Materials and methods

### Depositories

**NSMT** National Museum of Nature and Science (coll. Shibata), Tsukuba, Japan (S. Nomura)

**TARI** Taiwan Agricultural Research Institute, Taichung, Taiwan (C.-F. Lee)

### Specimen preparation and photography

The dry pinned specimens were examined using a Leica MZ APO Stereoscope. The aedeagi were extracted from the terminal segments (abdominal segments IX and X) and preserved in a microvial with glycerin, pinned with the specimens. The label information is given as written on the original labels, with slashes (/) used to indicate different labels. Photos were taken using a Canon EOS R7 camera with Canon RF 100 mm f/2.8L Macro IS USM Macro, Canon MP-E 65 mm 1–5× Marco, and LAOWA Aurogon FF 10–50× NA0.5 supermicro APO Macro lenses. Photos of the habitus, labrum, and mandibles were taken from dried, mounted specimens; photos of the palps, protarsi, abdominal segments, and aedeagus were taken under ethanol-based hand sanitizer. Images of specimens were stacked by HELICON FOCUS 8 and subsequently edited in PHOTOSHOP CS5. Habitat photos of *Luffy schillhammeri* sp. nov. were provided by Yu-Tang Wang and subsequently upscaled using TOPAZ GIGAPIXEL solely for resolution improvement and did not affect scientific interpretation.

### Comparative material

To assess the phylogenetic position of *Luffy* gen. nov., we examined specimens representing all genera of the *Ocypus*-group (Brunke and Smetana 2020), as well as the phylogenetically unresolved genera *Acupronotes*, *Apostenolinus*, and *Staphylinus*. For most of the genera we examined the type species. When type species were unavailable, we examined closely related species instead.

### Measurements

All measurements were taken with an eyepiece micrometer and given in mm. Their explanation and the abbreviations are as follows: Forebody length: from the base of the labrum to the posterior margin of elytra (**FL**); Whole body length: from the base of the labrum to the posterior margin of tergite VIII (**WL**); Eye length: maximum length of left eye (**EL**); Eye width: maximum width of eye (measured on left eye) (**EW**); Head length: distance from the base of the labrum to the posterior margin of head (**HL**); Head width: maximum width of head (**HW**); Temporal length: distance from the posterior margin of the eye to the neck constriction (**TL**); Pronotal length: distance from anterior margin of pronotum to the posterior margin of pronotum along midline (**PL**); Pronotal width: maximum width of the pronotum, located approximately at the anterior third (**PW**).

### Terminology

The morphological terminology for chaetotaxy is used in accordance with Zhao et al. (2025) as follows: **EDM**, elytral dorsal macrosetae; **ELM**, elytral lateral macrosetae; **FCP**, frontoclypeal punctures; **MTM**, middle tergal macrosetae; **PALM**, pronotal anterior-lateral macrosetae; **PAMM**, pronotal anterior-middle macrosetae; **PFM**, posterior frontal macrosetae; **PMM**, pronotal middle macrosetae; **POP**, parocular puncture with macrosetae; **PPM**, pronotal posterior macrosetae; **PsP**, parascutellar macrosetae; **PTM**, posterior tergal macrosetae; **TM**, temporal macrosetae.

Regarding the mandibular morphology of Staphylinina, we also follow the framework proposed by Zhao et al. (2025). However, since the terminology of mandibular structures in Zhao et al. (2025) was based solely on the left mandible and their homology and recognition were not sufficiently explained in that publication, here we refine it and give more details. In particular, Zhao et al. (2025) recognized the dorsal and ventral ridges (as well as the dorsal and ventral teeth located on the respective ridges) of the mandible but did not explain how to identify them. In the present study, the dorsal ridge is defined as a ridge that extends along the dorsal surface in the apical portion of the mandible, whereas the ventral ridge is the one that extends along the ventral surface of the apical portion of the mandible. These ridges may not be obvious in the middle and basal portions of a mandible, where the teeth are located. However, the teeth in these portions are usually connected to either dorsal or ventral ridges of the apical portion. Based on which ridge a given tooth is connected to, it is called dorsal or ventral ridge tooth, respectively. The basal extra tooth is recognized as an additional tooth posterior to the dorsal ridge tooth or ventral ridge tooth. However, its position is variable among different taxa, it may be located near the ventral ridge tooth or closer to the base of the mandible, and its homology among genera remains uncertain.

Following Zhao et al. (2025), the morphological terms for the discussed mandibular teeth are as follows: **BET**, basal extra tooth; **DRT**, dorsal ridge tooth. **VRT**, ventral ridge tooth. Given the importance of the mandibular ridges for determining the respective teeth homology, here we introduce two more abbreviations: **DR**, dorsal ridge; **VR**, ventral ridge, both normally best seen in the apical portion of the mandible, as discussed above.

## Taxonomy

### Genus Luffy gen. nov.

Taxon classificationAnimaliaColeopteraStaphylinidae

D28ED20E-E61F-593C-A359-34109B63BC81

https://zoobank.org/6EFBA1B1-CC01-41A0-B5A9-6F57B9CAA88B

**Figure 1.**
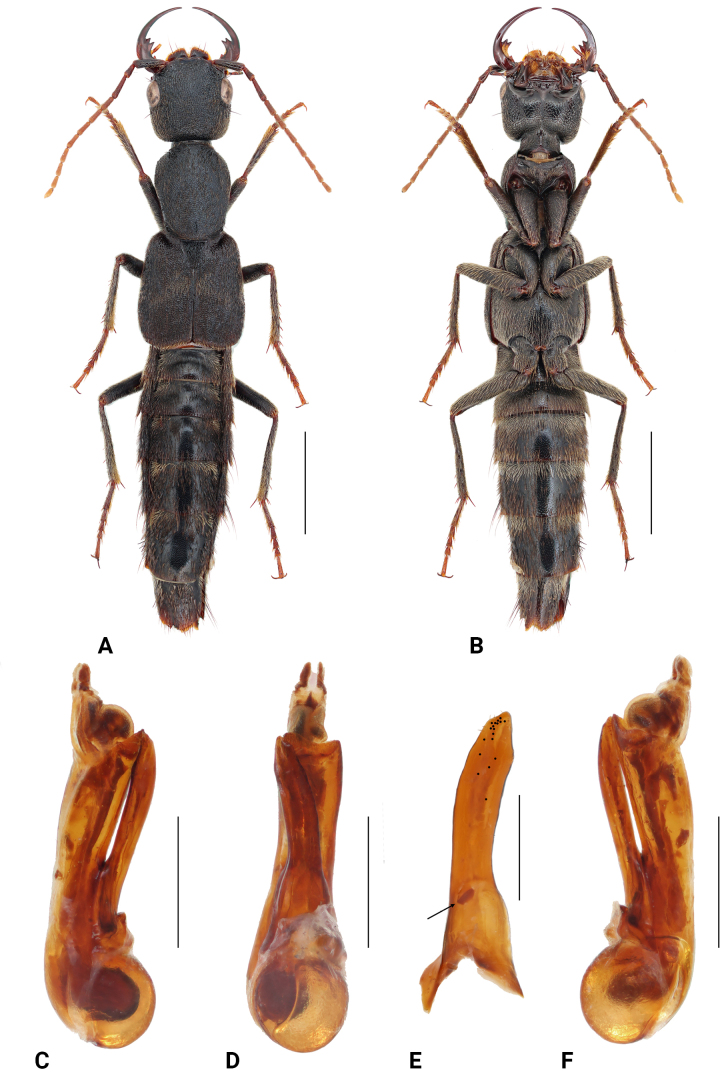
*Luffy schillhammeri* sp. nov., habitus and aedeagus. **A**. Habitus, dorsal view; **B**. Habitus, ventral view; **C, F**. Aedeagus, lateral view; **D**. Aedeagus, parameral view; **E**. Paramere, underside. The arrow indicates the articulation on paramere. The black dots on the paramere strengthen the position of the peg setae. Scale bars: 3 mm (**A, B**); 1 mm (**C–F**).

**Figure 2.**
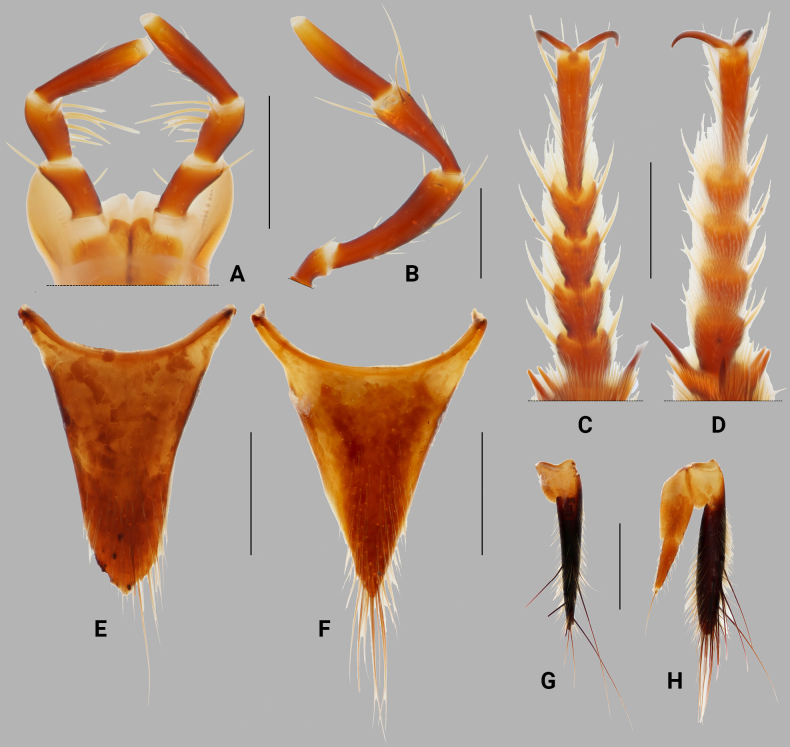
*Luffy schillhammeri* sp. nov., labium, maxillary palps, protarsi, abdominal segments IX–X. **A**. Labium; **B**. Maxillary palps; **C**. Protarsi, dorsal view; **D**. Protarsi, ventral view; **E**. Tergite X, male; **F**. Tergite X, female; **G**. Abdominal segment IX, male; **H**. Abdominal segment IX and gonocoxite, female. Scale bars: 1 mm.

**Figure 3.**
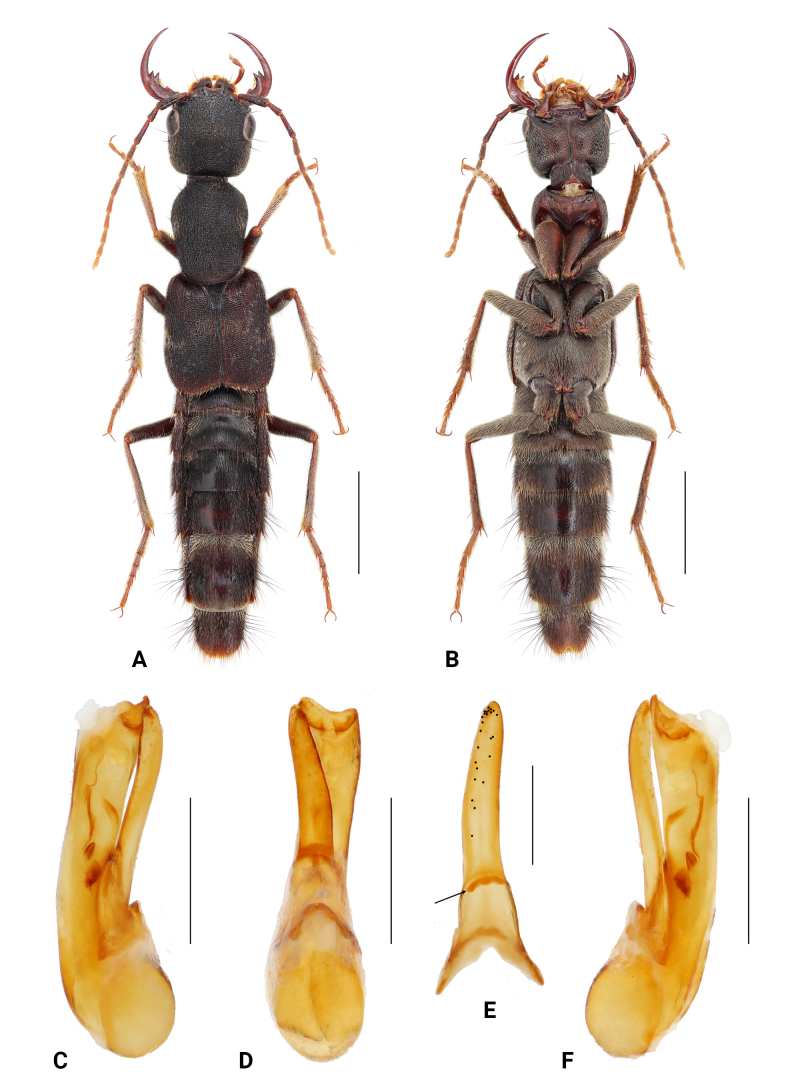
*Luffy nika* sp. nov., habitus and aedeagus. **A**. Habitus, dorsal view; **B**. Habitus, ventral view; **C, F**. Aedeagus, lateral view; **D**. Aedeagus, parameral view; **E**. Paramere, underside. The arrow indicates the articulation on paramere. The black dots on the paramere strengthen the position of the peg setae. Scale bars: 3 mm (**A, B**); 1 mm (**C–F**).

Figs 1, 2, 3, 5A, 6A

#### Type species:

*Luffy schillhammeri* sp. nov.

#### Included species:

*Luffy nika* sp. nov., *L. schillhammeri* sp. nov.

#### Diagnosis.

The genus can be diagnosed by narrow, not expanded protarsi in both sexes (Fig. 2C, D) and all antennomeres longer than wide (Figs 1A, 1B, 3A, 3B), both traits being unique synapomorphies within Staphylinina. In addition to these characters, the new genus can be further distinguished by the combination of the presence of semimembranous extension on the labrum (Fig. 5A) and the presence of DRT1, DRT2, and BET on the left mandible (Fig. 6A).

**Figure 4.**
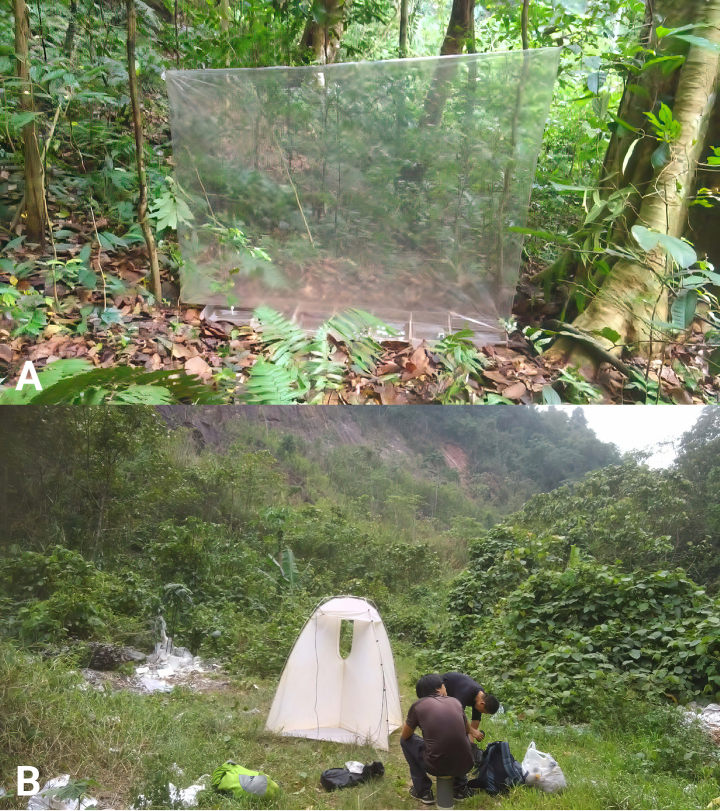
Habitats of *Luffy schillhammeri* sp. nov. in Yunnan, China. **A**. Manzhang (曼掌); **B**. Manfeng (曼粉) (Photos by Y.-T. Wang).

**Figure 5.**
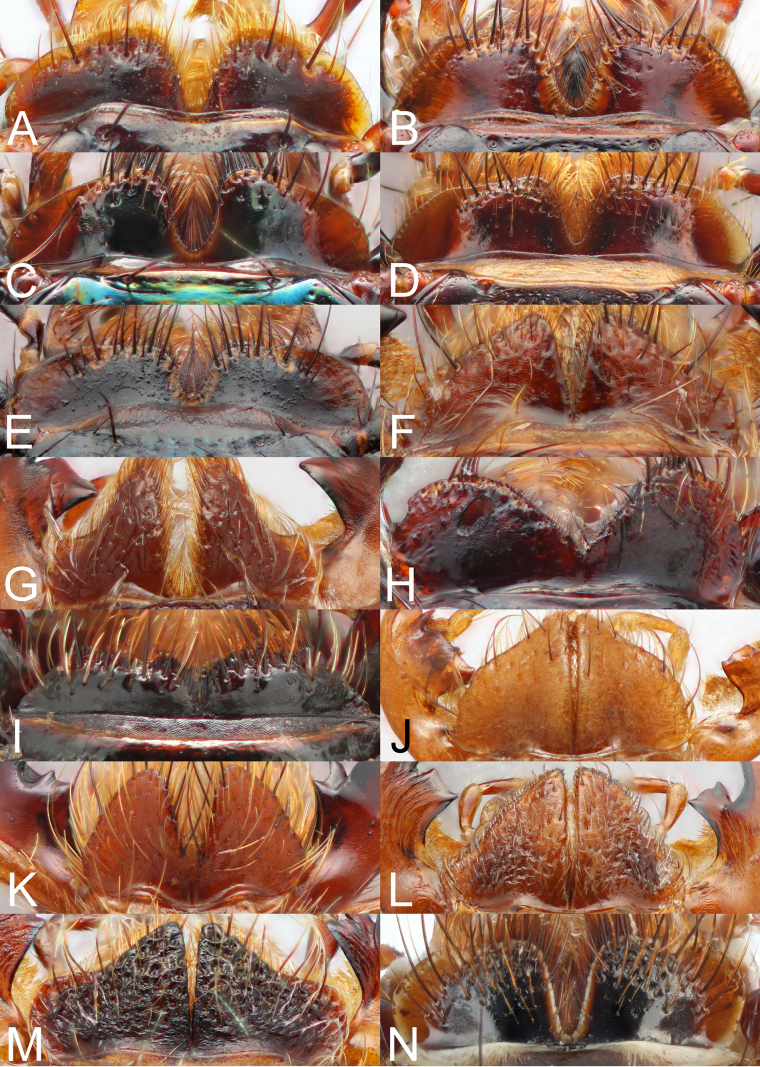
Diversity of labrum morphology of *Ocypus*-group and *Staphylinus*. **A**. *Luffy*; **B**. *Agelosus*; **C**. *Cyanocypus*; **D**. *Protocypus*; **E**. *Ocypus*; **F**. *Eucibdelus*; **G**. *Guillaumius*; **H**. *Menoedius*; **I**. *Parapalaestrinus*; **J**. *Paraphytolinus*; **K**. *Rhynchocheilus*; **L**. *Rhyncocheilus*; **M**. *Trichocosmetes*; **N**. *Staphylinus*.

**Figure 6.**
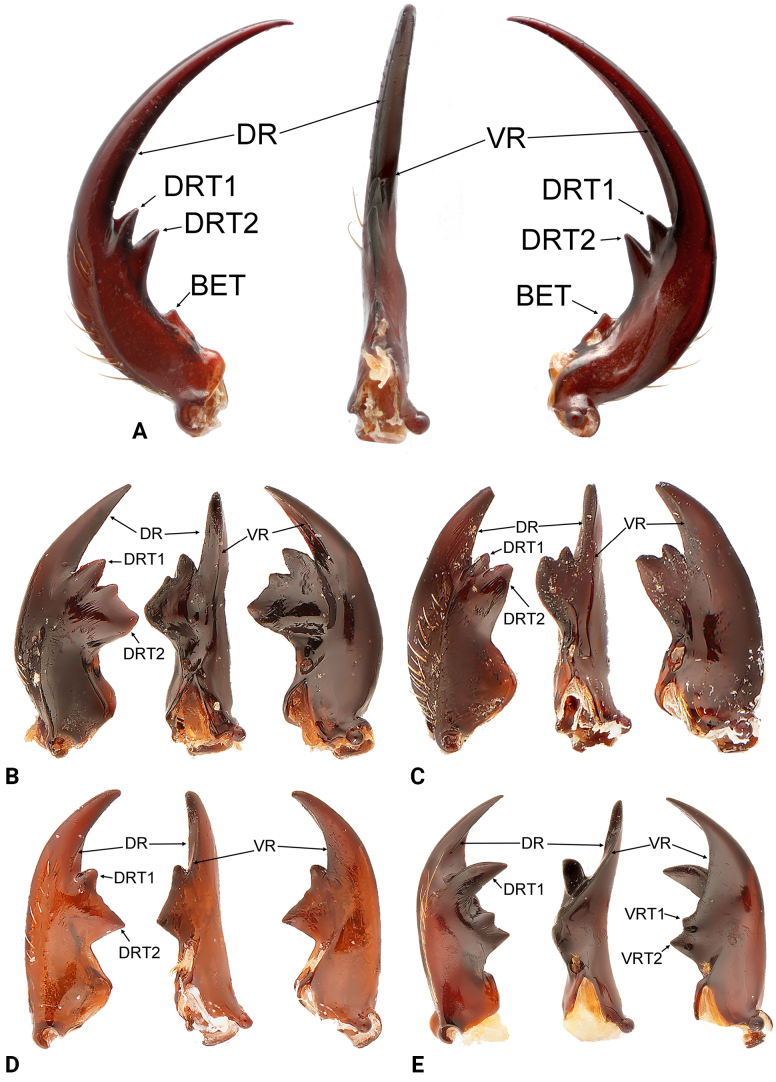
Mandibular morphology of *Luffy* and *Eucibdelus* lineage. **A**. *Luffy*; **B**. *Menoedius*; **C**. *Parapalaestrinus*; **D**. *Eucibdelus*; **E**. *Rhynchocheilus* (Photos A by the authors; photos B-E by Q.-H. Zhao). Abbreviations: Abbreviations: BET: basal extra tooth; DR: dorsal ridge; DRT: dorsal ridge teeth; VR: ventral ridge; VRT: ventral ridge teeth.

#### Description.

Medium-sized Staphylinina (TL: 16.4–20.5 mm; FL: 8.4–10.3 mm).

***Head***. Head slightly transverse (HL/HW = 0.8). Tempora not expanded in both sexes. Eyes large and convex, slightly shorter than tempora (EL/TL: 0.7–0.8). Head densely punctate on both dorsal and ventral faces, punctures rounded to irregular. Labrum moderately large, trapezoidal, with distinct semimembranous margin (Fig. 5A); anterior portion with two macrosetae. Gular sutures not separated. Antennae very long, all antennomeres longer than wide, with dense pubescence. Mentum with two pairs of macrosetae located in the anterior portion; two pairs of thinner setae located at the anterior margin. Labial palps long; last segment setose, longer than the first two segments (Fig. 2A). Maxillary palps long; first segment short; second segment as long as the third and last segments combined; last segment with very minute setae, almost unobservable even at 50× magnification (Fig. 2B). Mandibles very long and slender, both left and right mandibles with DRT1, DRT2 and BET, DRT1, and DRT2 forming a larger bidentate tooth; BET close to the base of mandible on both sides of mandible, located above the prostheca (Fig. 6A). Prostheca single-lobed. Head chaetotaxy: FCP present; TM close to posterior margin of eyes; 1 POP present; PFM missing.

***Thorax***. Pronotum pyriform, longer than wide (PL/PW: 1.32–1.41), densely punctate; impunctate midline almost invisible. Anterior angles rounded. Superior and inferior marginal lines join each other approximately one-third from the anterior margins; pronotal hypomera asetose. Pronotum chaetotaxy: PAMM1 present, PALM absent, PMM present, located at approximately one-third of pronotal length from the anterior margin. Mesoventrite ridge absent. Elytra and hind wings fully developed, elytra with a distinct transverse curved stripe of pale setae in both species. Elytral chaetotaxy: EDM1, 2, 3 present; PsP2 absent. Protibiae slender, without modification, with 2–3 spines on lateral face; meso-and metatibiae straight, not arcuate, mesotibiae with numerous spines; metatibiae with 2–3 spines on lateral face. Protarsi not expanded; underside densely setose, but setae unmodified (Fig. 2C, D).

***Abdomen***. All segments densely covered with long setae. Tergites III–V with distinct accessory ridges, with paler setal patches besides the outer margin of accessory ridges; tergites VI and VII with paler setae in anterior margin. Tergite VII with a distinct pale apical seam of palisade setae. Abdominal segment VII longer than other segments. Abdominal segment chaetotaxy: MTM and PTM present; segments VII and VIII with numerous macrosetae.

**Male**. Sternite VIII with a moderately deep, subtriangular emargination at midpoint of posterior margin. Abdominal segment IX clavate (Fig. 2G). Tergite X triangular (Fig. 2E). Aedeagus strongly asymmetrical; paramere slightly bent to the left side in parameral view; peg setae present in the anterior portion but not strongly pigmented.

**Female**. The only known female of *L. schillhammeri* sp. nov. is slightly larger than males of both species. Abdominal segment IX clavate, slightly wider than in male (Fig. 2H). Tergite X triangular (Fig. 2F), fully sclerotized, without a membranous part.

#### Etymology.

The generic name *Luffy* is the given name of the main character of the famous manga “One Piece”, Monkey D. Luffy, who has the ability to extend his body parts. It refers to the elongated antennae, palps, and mandibles of the new genus. The gender is masculine.

### Luffy schillhammeri sp. nov.

Taxon classificationAnimaliaColeopteraStaphylinidae

7FEA42F8-FB6E-5FE1-A137-058F2CAF2EA8

https://zoobank.org/60E461D7-36C4-4CBA-86B4-A49FDA1CFAF3

Figs 1, 2

#### Material examined.

***Holotype***: • male, **China**: Yunnan, CCCC Manzhang (曼掌), 27.III.2018. leg. Y.-T. Wang / Mengla County, 21.9266, 101.1951, alt. 650 m, FIT, primary and secondary broadleaf forests (TARI).

***Paratype***: • 1 female, **China**: Yunnan, CCCC Manfeng (曼粉), 12.V.2016. leg. Y.-T. Wang / Mengla County, 21.4066, 101.6312, alt. 750 m, light trap, primary and secondary broadleaf forests / Project: Revision of *Ocypus* group DNA Voucher specimen: FS363 (TARI).

#### Diagnosis and comparison.

*Luffy schillhammeri* sp. nov. is very similar to *L. nika* sp. nov. It can be distinguished from *L. nika* sp. nov. by differences in pronotal shape: *L. schillhammeri* sp. nov. has a slightly shorter pronotum (PL/PW = 1.32), gradually narrowing basad from the anterior third of its length (Fig. 1A), whereas *L. nika* sp. nov. has a slightly longer pronotum (PL/PW = 1.41), more distinctly narrowing basad (Fig. 3A). More reliable identification of these species requires examination of the aedeagus: *L. schillhammeri* sp. nov. has a shallower emargination of the median lobe (Fig. 1D), a wider paramere (Fig. 1D, E), a subtriangular parameral apex (Fig. 1D, E), and (on the underside) a small asymmetrical area of its articulation to the median lobe (Fig. 1E); whereas *L. nika* sp. nov. has a deeper apical emargination of the median lobe (Fig. 3D), a thinner paramere (Fig. 3D, E), a rounded parameral apex (Fig. 3E), and (on the underside) a transverse, more symmetrical area of its articulation to the median lobe (Fig. 3E).

#### Description.

Measurements (given in mm): Male, WL: 16.79; FL: 8.4; EL: 0.7; EW: 0.3; HL: 2.4; HW: 2.6; TL: 1; PL: 2.9; PW: 2.2. Female, WL: 20.48; FL: 10.25; EL: 0.8; EW: 0.4; HL: 2.7; HW: 2.9; TL: 1.1; PL: 3.3; PW: 2.5.

***Habitus*** (Figs 1, 2). Body black, without metallic lustre. Head, pronotum and elytra densely punctate with long black setae, punctures mostly rounded, more irregular on vertex area.

***Head*** slightly transverse, without impunctate midline. Eyes large and protruding. Antennae rufotestaceous to black, first five antennomeres darker than others; all antennomeres distinctly longer than wide. Head with patches of white setae on tempora, with sparse white setae on disc.

***Pronotum*** pyriform, longer than wide, gradually narrowing basad from one-third of pronotal length; densely punctate, with rounded punctures; with small patches of white setae at midlength of lateral margin; without impunctate midline. Elytra and scutellum densely covered with black setae; elytra with a distinct, transverse, curved stripe of white setae. Legs covered with dense white setae.

***Abdomen*** tergites black; tergites III–V each with accessory basal ridges. Tergites III–VII densely covered with long, black setae; tergites III–V each with paired patches of white setae antero-laterally, extending to paratergites; tergites VI–VII with transverse stripe of white setae across anterior margin; tergite VIII with mixture of white and black setae. Sternites III–VIII black; sternites III–IV densely covered with white setae; sternite V with white setae on anterior and posterior margins, with brown setae in the middle portion; sternites VI–VIII with white setae on anterior portion and brown setae in the posterior portion.

**Male** sternite VIII with a moderately deep, subtriangular emargination at midpoint of posterior margin. Aedeagus slender, asymmetrical; median lobe parallel-sided in basal portion in parameral view, apex with a shallow emargination (Fig. 1C–F). Paramere moderately wide, slightly twisted to the left, apex subtriangular; underside with rather sparse and not strongly pigmented peg setae forming an apical cluster, with a few additional ones sparse in anterior portion (Fig. 1C–F). Underside of paramere with a distinct asymmetrical area of articulation to the median lobe (Fig. 1E).

**Female** slightly larger and more robust than male, but otherwise no marked sexual dimorphism.

#### Etymology.

The species is named in honour of Harald Schillhammer (Vienna, Austria), in recognition of his decades of contributions to the taxonomy of Staphylininae.

#### Distribution.

The species is known from the type locality in Mengla County, southwestern Yunnan Province, China.

#### Ecology.

*Luffy schillhammeri* sp. nov. was collected in low-altitude primary and secondary broadleaf forests (Fig. 4) by flight-intercept and light traps.

### Luffy nika sp. nov.

Taxon classificationAnimaliaColeopteraStaphylinidae

961DFFD6-1739-5FFF-8024-02D6F7C59CB8

https://zoobank.org/F0E1DA34-2544-4A36-B2F2-12E412B94F53

Fig. 3

#### Material examined.

***Holotype***: • male, **Laos**, NW., Kiolom, 1050 m alt., 36 km NW from L N. Louang Namtha, 11.V.2004, Y. Shibata leg. / NSMT-I-C Shibata C No. SBC006913 (NSMT).

***Paratype***: • 1 male, same data as the holotype, but NSMT-I-C Shibata C No. SBC006914 (NSMT).

#### Diagnosis and comparison.

*Luffy nika* sp. nov. is very similar to *L. schillhammeri* sp. nov. It can be distinguished from *L. schillhammeri* sp. nov. by differences in pronotal shape: *L. nika* sp. nov. has a slightly longer pronotum (PL/PW = 1.41), distinctly narrowing basad from the anterior third of its length (Fig. 3A), whereas *L. schillhammeri* sp. nov. has a slightly shorter and more gradually narrowing pronotum (PL/PW = 1.32) (Fig. 1A). More reliable identification of the two species requires examination of the aedeagus: *L. nika* sp. nov. has a deeper apical emargination of the median lobe (Fig. 3D), a thinner paramere (Fig. 3D, E) with a rounded apex (Fig. 3E), and a more symmetrical transverse area of its articulation to the median lobe (Fig. 3E); whereas *L. schillhammeri* sp. nov. has a shallower apical emargination of the median lobe (Fig. 1D), and a wider paramere (Fig. 1D, E) with a subtriangular apex (Fig. 1E), and a distinct asymmetrical area of its articulation to the median lobe (Fig. 1E).

#### Description.

Measurements (given in mm, average provided in brackets): Male, WL: 16.39–17.36 (16.88); FL: 8.64–8.95 (8.8); EL: 0.8 (0.8); EW: 0.4 (0.4); HL: 2.5 (average 2.5); HW: 2.7 (2.7); TL: 1 (1); PL: 3.1 (3.1); PW: 2.2 (2.2).

***Habitus*** (Fig. 3A, B). Body black, without metallic lustre. Head, pronotum and elytra densely punctate with long black setae; punctures mostly rounded, more irregular on vertex area.

***Head*** slightly transverse, without impunctate midline. Eyes large and protruding. Antennae rufotestaceous to black, first five antennomeres darker than others; all antennomeres distinctly longer than wide. Head with patches of white setae on tempora, with sparse white setae on disc.

***Pronotum*** pyriform, longer than wide, distinctly narrowing basad from one-third of its length; densely punctate, with rounded punctures; with small patches of white setae at midlength; without impunctate midline. Elytra and scutellum densely covered with black setae; elytra with a distinct, transverse, curved stripe of white setae. Legs covered with dense white setae.

***Abdomen*** tergites black; tergites III–V each with accessory basal ridges. Tergites III–VII densely covered with long, black setae; tergites III–V each with paired patches of white setae antero-laterally, extending to paratergites; tergites VI–VII with transverse stripe of white setae across anterior margin; tergite VIII with mixture of white and black setae. Sternites III–VIII black; sternites III–IV densely covered with white setae; sternite V with white setae on anterior and posterior margins, with brown setae in the middle portion; sternites VI–VIII with white setae in the anterior portion and brown setae in the posterior portion.

**Male** sternite VIII with a moderately deep, subtriangular emargination at midpoint of posterior margin. Aedeagus slender, asymmetrical; median lobe parallel-sided in basal portion in parameral view, apex with a shallow emargination (Fig. 3C–F). Paramere thin, slightly twisted to the left, apex rounded; underside with rather sparse and not strongly pigmented peg setae forming an apical cluster, with a few additional ones sparse in anterior portion (Fig. 3C–F). Underside of paramere with a distinct, narrow, transverse symmetrical area of its articulation to the median lobe (Fig. 3F).

**Female** unknown.

#### Etymology.

The specific epithet *nika* is derived from the Devil Fruit of the main character, Luffy, in the famous manga *One Piece*, in reference to the genus name. It also alludes to the pale hairs of the new species, reminiscent of Luffy’s “Nika” form, in which his clothing and hair turn white. It is a noun in apposition.

#### Ecology.

Nothing is known about the habitat or collecting circumstances for *L. nika* sp. nov.

#### Distribution.

The species is known from the type locality in Louang Namtha in northwestern Laos.

#### Remarks.

The terminal segment of the maxillary and labial palps in both specimens of *L. nika* sp. nov. bears a protruding membranous structure, although it is less pronounced in one specimen. We are uncertain whether this represents a species-specific character or an artefact resulting from specimen preparation. Therefore, this character was not included in the diagnosis, pending examination of additional material in the future.

## Discussion

### *Luffy* as a member of the *Ocypus*-group

The genus *Luffy* gen. nov. can be undoubtedly placed within the *Ocypus*-group of the subtribe Staphylinina. This placement is supported by several symapomorphies which were proposed by earlier authors (Brunke and Smetana 2019, 2020; Zhao et al. 2025): the presence of the PAMM2 on the pronotum, the absence of PsP2 on the elytra, and the characteristic asymmetrical aedeagus. The presence of PAMM2, lack of PsP2, and the presence of DRT on the left mandible in *Luffy* strongly suggest its close relationship with the *Eucibdelus* lineage which is further discussed below.

### *Luffy* and labral synapomorphy of the *Eucibdelus* lineage

Schillhammer (2023) defined the *Eucibdelus* lineage primarily by the absence of a semimembranous extension on the labrum (resulting in a fully sclerotized labrum) and the frequent occurrence of dilated protibiae and patellate protarsi. Our investigation corroborates the stability of the labrum morphology within the *Eucibdelus* lineage where all examined taxa possess a fully sclerotized labrum (Fig. 5F–M), whereas other members of the *Ocypus* group and the genus *Staphylinus* exhibit at least a thin semimembranous portion along lateral margins of labrum (Fig. 5B–E, N). In contrast, other characters previously used to diagnose the *Eucibdelus* lineage show a significant degree of variation. For example, among the species we examined, protibial dilation ranges from extremely dilated (e.g. *Rhynchocheilus popeye*; Schillhammer 2025: fig. 5) to entirely slender protibiae (e.g. the genus *Sphaeromacrops*; Tang and Cheng 2020: fig. 1). Such variation is even present within a single genus, likely reflecting different hunting strategies (e.g. the dilated protibiae of *R. aureus* versus the slender protibiae of *R. dohertyi*; Schillhammer 2012: figs 1, 3). In addition, the shape of the protarsal structures, that is the degree to which the protarsi are “patellate”, varies across genera and species too. Because this is a continuous character, it is often difficult to break it into discrete states. This observed variation is also consistent with the variation noted in Schillhammer (2023). Like the members of the *Eucibdelus* lineage, *Luffy* has a large and heavily sclerotized labrum which, however, retains a clearly semimembranous lateral margin (Fig. 5A), consistent with other members of the *Ocypus* group (Fig. 5B–E).

### *Luffy* and a new mandibular synapomorphy of the *Eucibdelus* lineage

Through extensive morphological comparison, we identified a novel synapomorphy for the *Eucibdelus* lineage: the presence of a DRT on the left mandible (Fig. 6B–E). Based on a fully sclerotized labrum and the presence of the DRT on the left mandible, the *Eucibdelus* lineage seems to represent a monophylum that includes the following genera: *Eucibdelus* Kraatz; *Guillaumius* Schillhammer; *Menoedius* Fauvel; *Palaestrinus* Erichson; *Parapalaestrinus* Bernhauer; *Paraphytolinus* Hayashi; *Phytolinus* Sharp; *Rhynchocheilus* Sharp; *Rhyncocheilus* Fauvel; *Sphaeromacrops* Schillhammer; *Trichocosmetes* Kraatz.

**Figure 7.**
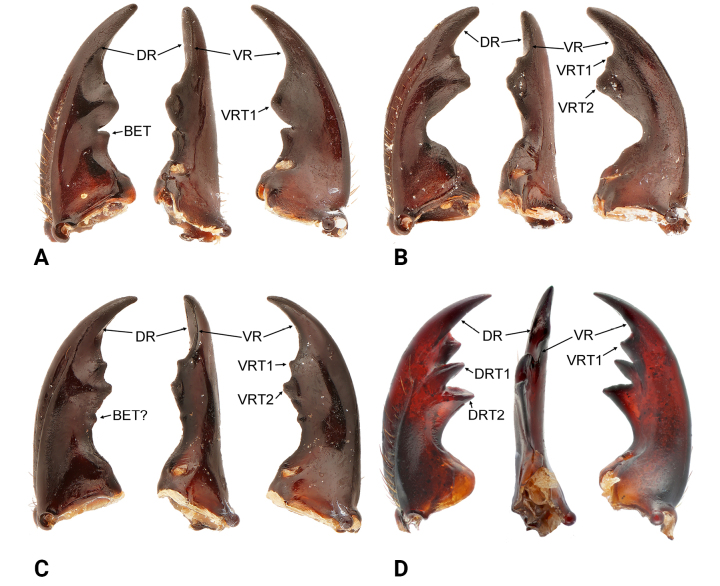
Diversity of mandibular morphology of the left teeth of *Ocypus*-group and *Staphylinus*. **A**. *Agelosus*; **B**. *Protocypus*; **C**. *Ocypus*; **D**. *Staphylinus* (Photos A–C by Q.-H. Zhao; photo D by the authors). Abbreviations: Abbreviations: BET: basal extra tooth; DR: dorsal ridge; DRT: dorsal ridge teeth; VR: ventral ridge; VRT: ventral ridge teeth.

Interestingly, within all other Staphylinina only *Luffy* (Fig. 7A) and *Staphylinus* (Fig. 7D) have the DRT. The systematic position of *Staphylinus* remains contentious (Brunke and Smetana 2020). Although it shares the asymmetrical aedeagus and other traits with the *Ocypus*-group, lack of PAMM2 and presence of PsP2 in this genus align it with the *Platydracus*-group. This suggests that *Staphylinus* may represent an intermediate taxon between two groups, while the presence of the DRT in this genus and in the *Eucibdelus* lineage could be a result of homoplasy.

### Systematic position of *Luffy*

Like the discussed genus *Staphylinus*, *Luffy* is a phylogenetically peculiar genus too. The presence of PAMM2, lack of PsP2, and the presence of DRT on the left mandible in *Luffy* strongly suggest its placement in the *Eucibdelus* lineage of the *Ocypus*-group. However, clearly semimembranous lateral margins of the labrum in *Luffy*, a character state (Fig. 5A) shared with all other members of the *Ocypus*-group (Fig. 5B–E) except the *Eucibdelus* lineage, suggests its position as a sister group to the latter and an evolutionary link from more plesiomorphic members of the *Ocypus*-group to their more specialized clade forming the *Eucibdelus* lineage. On the other hand, *Luffy* presents several unique autapomorphies of its own, such as non-dilated protarsi (Fig. 2C, D) and all antennomeres being longer than wide (Figs 1A, 1B, 3A, 3B), suggesting its own special adaptation to a microhabitat and biology which is yet unknown.

## Supplementary Material

XML Treatment for Luffy

XML Treatment for Luffy schillhammeri

XML Treatment for Luffy nika

## Additional information

### Artificial Intelligence (AI) use

The authors accept full responsibility for the content of the manuscript, including the disclosure of any use of AI.

Regarding the use of AI in preparing this manuscript, the authors declare the following: We only used Topaz Gigapixel AI for resolution improvement and did not affect scientific interpretation, as explained in the materials and methods.

**Used for**: Language, style and writing. The ChatGPT 5.5 and Gemini 3.5 Flash were used to check the grammar and the structure of the texts. But the entire text was largely revised by us after AI review.

### Author ORCIDs

F.-S. Hu https://orcid.org/0000-0003-1269-8904

A. Solodovnikov https://orcid.org/0000-0003-2031-849X

### Data availability

All of the data that support the findings of this study are available in the main text.
